# 4494 Predictors of Reintubation After Cardiac Surgery

**DOI:** 10.1017/cts.2020.182

**Published:** 2020-07-29

**Authors:** Robert Edward Freundlich, Gen Li, Jonathan P Wanderer, Frederic T Billings, Henry Domenico, Daniel Byrne, Pratik Pandharipande

**Affiliations:** 1 Vanderbilt University Medical Center

## Abstract

OBJECTIVES/GOALS: We modeled risk of reintubation within 48 hours of cardiac surgery using variables available in the electronic health record (EHR). This model will guide recruitment for a prospective, pragmatic clinical trial entirely embedded within the EHR among those at high risk of reintubation. METHODS/STUDY POPULATION: All adult patients admitted to the cardiac intensive care unit following cardiac surgery involving thoracotomy or sternotomy were eligible for inclusion. Data were obtained from operational and analytical databases integrated into the Epic EHR, as well as institutional and departmental-derived data warehouses, using structured query language. Variables were screened for inclusion in the model based on clinical relevance, availability in the EHR as structured data, and likelihood of timely documentation during routine clinical care, in the hopes of obtaining a maximally-pragmatic model. RESULTS/ANTICIPATED RESULTS: A total of 2325 patients met inclusion criteria between November 2, 2017 and November 2, 2019. Of these patients, 68.4% were male. Median age was 63.0. The primary outcome of reintubation occurred in 112/2325 (4.8%) of patients within 48 hours and 177/2325 (7.6%) at any point in the subsequent hospital encounter. Univariate screening and iterative model development revealed numerous strong candidate predictors (ANOVA plot, figure 1), resulting in a model with acceptable calibration (calibration plot, figure 2), c = 0.666. DISCUSSION/SIGNIFICANCE OF IMPACT: Reintubation is common after cardiac surgery. Risk factors are available in the EHR. We are integrating this model into the EHR to support real-time risk estimation and to recruit and randomize high-risk patients into a clinical trial comparing post-extubation high flow nasal cannula with usual care. CONFLICT OF INTEREST DESCRIPTION: REF has received grant funding and consulting fees from Medtronic for research on inpatient monitoring.

